# Pathological mechanisms of neuroimmune response and multitarget disease-modifying therapies of mesenchymal stem cells in Parkinson’s disease

**DOI:** 10.1186/s13287-023-03280-0

**Published:** 2023-04-12

**Authors:** Yi Zhuo, Xuan Li, Zhengwen He, Ming Lu

**Affiliations:** 1 Department of Neurosurgery, The Affiliated Cancer Hospital of Xiangya School of Medicine, Central South University/Hunan Cancer Hospital, Changsha, 410000 Hunan China; 2 The National and Local Joint Engineering Laboratory of Animal Peptide Drug Development, College of Life Sciences, Hunan Normal University, Changsha, 410006 Hunan China; 3 Hunan Provincial Key Laboratory of Neurorestoratology, The Second Affiliated Hospital (the 921st Hospital of PLA), Hunan Normal University, Changsha, 410003 Hunan China

**Keywords:** Parkinson’s disease, Neuroimmune response, Mesenchymal stem cells, Immunotherapy

## Abstract

Parkinson's disease (PD) is a neurodegenerative disease characterized by the degeneration of dopaminergic neurons in the substantia nigra (SN); the etiology and pathological mechanism of the disease are still unclear. Recent studies have shown that the activation of a neuroimmune response plays a key role in the development of PD. Alpha-synuclein (α-Syn), the primary pathological marker of PD, can gather in the SN and trigger a neuroinflammatory response by activating microglia which can further activate the dopaminergic neuron’s neuroimmune response mediated by reactive T cells through antigen presentation. It has been shown that adaptive immunity and antigen presentation processes are involved in the process of PD and further research on the neuroimmune response mechanism may open new methods for its prevention and therapy. While current therapeutic regimens are still focused on controlling clinical symptoms, applications such as immunoregulatory strategies can delay the symptoms and the process of neurodegeneration. In this review, we summarized the progression of the neuroimmune response in PD based on recent studies and focused on the use of mesenchymal stem cell (MSC) therapy and challenges as a strategy of disease-modifying therapy with multiple targets.

## Introduction

Parkinson's disease (PD) is a neurodegenerative disease characterized by the loss of dopaminergic neurons in the substantia nigra [1, 2]. The main clinical manifestations are quiescent tremors, myotonia, tardiness, and abnormal postural gait [3, 4]. Abnormalities of the immune system are also considered an essential component of PD susceptibility and progression, and this area of research has received increasing attention over the past decade. PD is characterized by the death of dopaminergic neurons containing Lewy bodies (LB) composed mainly of alpha-synuclein (α-Syn) in the substantia nigra (SN), which provide the final diagnostic and pathological features for postmortem examinations [5, 6]. The α-Syn protein is a small acidic synaptic protein made up of 140 amino acids with a tendency to misfold and aggregate [7]. During the course of the disease, the major constituent of LB is misfolded α-Syn, which spreads to different brain regions in a prion-like fashion [8]. Microglia are the first immune defense system of the human brain and one of the main cell types involved in the inflammatory response of the central nervous system [9, 10]. The misfolded α-Syn protein can bind to and activate microglial cells through receptors on the surface of microglial cells, leading to a series of persistent inflammatory responses [11, 12].

Due to the lack of understanding of the cause of PD, symptomatic clinical therapy has traditionally focused on using medicines like levodopa and brain surgery. Besides the adverse effects associated with using long-term oral levodopa, drug resistance is the critical reason that renders the treatment ineffective. While they ameliorate patients' symptoms to some extent, surgeries like neuronuclear destruction and deep brain stimulation can cause irreversible neurological damage [13, 14]. In recent years, some vaccines and antibody formulations targeting α-Syn by active and passive immunization have been developed according to the main pathological markers of PD, and preliminary efficacy and safety assessments have been achieved [15–17]. Nerve repair, replacement, and regeneration have become possible with the development of stem cell technology and progress has been made in treating PD with stem cell transplantation. Mesenchymal stem cells (MSCs) are adult stem cells with paracrine, immune regulation, and multidirectional differentiation potential [18–20]. It was previously believed that the main pathogenic mechanism of PD was the loss of dopaminergic neurons and most researchers considered that the therapeutic effect of MSCs on PD was attributable to their cell replacement ability [21, 22]. The research focus of MSCs has shifted from cell replacement to multitarget therapy such as paracrine and immune regulation [23, 24]. The results of the studies also show that MSCs can accelerate the clearance and degradation of α-Syn through multitarget-modifying, such as modulating microglia activation, autophagic pathway, endocytosis, and protease secretion [25–28]. Research in the emerging field of PD immunomodulation provides an opportunity to identify new therapeutic targets and strategies to attenuate or reverse neurodegenerative changes. This review provides a concise overview of the development of neuroimmune responses and immune regulations in PD vaccine and antibody preparations targeting α-Syn through active and passive immunization, and the latest theoretical research and efficacy evaluation results of MSCs for the treatment of PD through multitarget disease modifications.

## The mechanism of the neuroimmune response in PD

### The α-Syn prion-like spreading

The aggregation of misfolded proteins in the central nervous system is a crucial hallmark of several age-related neurodegenerative diseases, including PD, Alzheimer's disease, and amyotrophic lateral sclerosis [29]. These diseases share key biochemical and biophysical characteristics with prion diseases. PD patients are believed to have a neuropathological basis denoted by the presence of LBs, which mostly comprise α-Syn inclusions. Pathological aggregation of α-Syn and its propagation through synaptic coupling are increasingly recognized as the basis for the pathophysiological progression of PD and related synuclein diseases [29]. Although the precise molecular mechanisms responsible for pathological accumulation and diffusion of α-Syn in the central nervous system are unclear, there is increasing evidence that misfolding and/or neuronal internalization of α-Syn promotes conformational template formation of endogenous α-Syn in monocytes by mechanisms similar to prion formation [30]. Microglia are macrophage-like populations of the central nervous system, which remain quiescent until injury or infection activates the cells to perform inflammatory functions and antigen-presenting cell (APC) functions [31]. The α-Syn aggregates in the central nervous system and can trigger a neuroinflammatory response by activating microglia, which can further activate the dopaminergic neuron neuroimmune response mediated by reactive T cells through antigen presentation [4, 31, 32].

### Innate and adaptive immunity

There are innate and adaptive parts of the immune system that work together to fight infection; however, when the immune system is maladjusted, the immune response can be a significant trigger of the disease. The innate immune system is the body’s first line of defense against the invasion of pathogens and consists of various cells that perform functions such as phagocytosis and antigen presentation. Due to the blood–brain barrier (BBB), the human brain has long been considered an immune-privileged organ. Prior research on the immune response of neurodegenerative diseases mainly focused on the innate immune system including dendritic cells, macrophages, and microglia, which can be activated by recognizing pathogen-associated molecular patterns and pattern-recognition receptors of endogenous damage-associated molecular patterns (such as α-Syn) [33–35]. The adaptive immune system of B and T lymphocytes produces a highly specific, targeted response to a wide variety of intracellular and extracellular infections [36]. CD4 + or CD8 + T cells are naive until their T cell receptors (TCRs) recognize the specific antigen presented by antigen-presenting cells via major histocompatibility complex (MHC) molecules [37]. In general, endogenous antigens are presented to CD8 + T cells by MHC-I, while exogenous resistance components are presented to CD4 + T cells by MHC-II [38]. All cells in the human body express MHC-I and can activate CD8 + T cells, while only specialized antigen-presenting cells including dendritic cells, macrophages, and microglia express MHC-II and have the ability to activate CD4 + T cells [38]. Once activated in secondary lymphoid organs, naive T cells differentiate into effector cells with specific functions to adapt to infection. CD8 + T cells can differentiate into cytotoxic T lymphocytes and induce apoptosis of infected cells without affecting adjacent healthy cells [39]. CD4 + T cells differentiate into helper T cell subtypes (Th1, Th2, and Th17 cells), which can produce various cytokines that help B lymphocytes [39]. Once activated by their specific antigen, primitive B lymphocytes can differentiate into plasma cells with the help of T helper cells, which produce antibodies specific to the antigen and promote clearance through phagocytosis [39].

The recent discovery of meningeal lymphatic vessels has directly overturned the traditional idea of brain immune privilege [40, 41]. There is evidence that central nervous system antigens can interact with lymphocytes and antigen-presenting cells through meningeal lymphatic pathways, which suggests that both innate and adaptive immune responses may be involved in the development of neurodegenerative disease [40–42]. Many studies have shown that persistent inflammatory response, microglial cell activation, and T cell infiltration are common characteristics of PD models and patients, and these factors play a vital role in the degeneration of dopaminergic neurons [43–45]. Seeking new therapeutic strategies to suppress neuroinflammatory responses can prevent or delay the loss of neurons in the SN and prevent disease progression. We have delineated an overview of the development of the neuroimmune response to PD as provided below.

## Mechanisms and roles of α-Syn in microglia cells

### PD and brain–gut–microbe axis

**Fig. 1:**
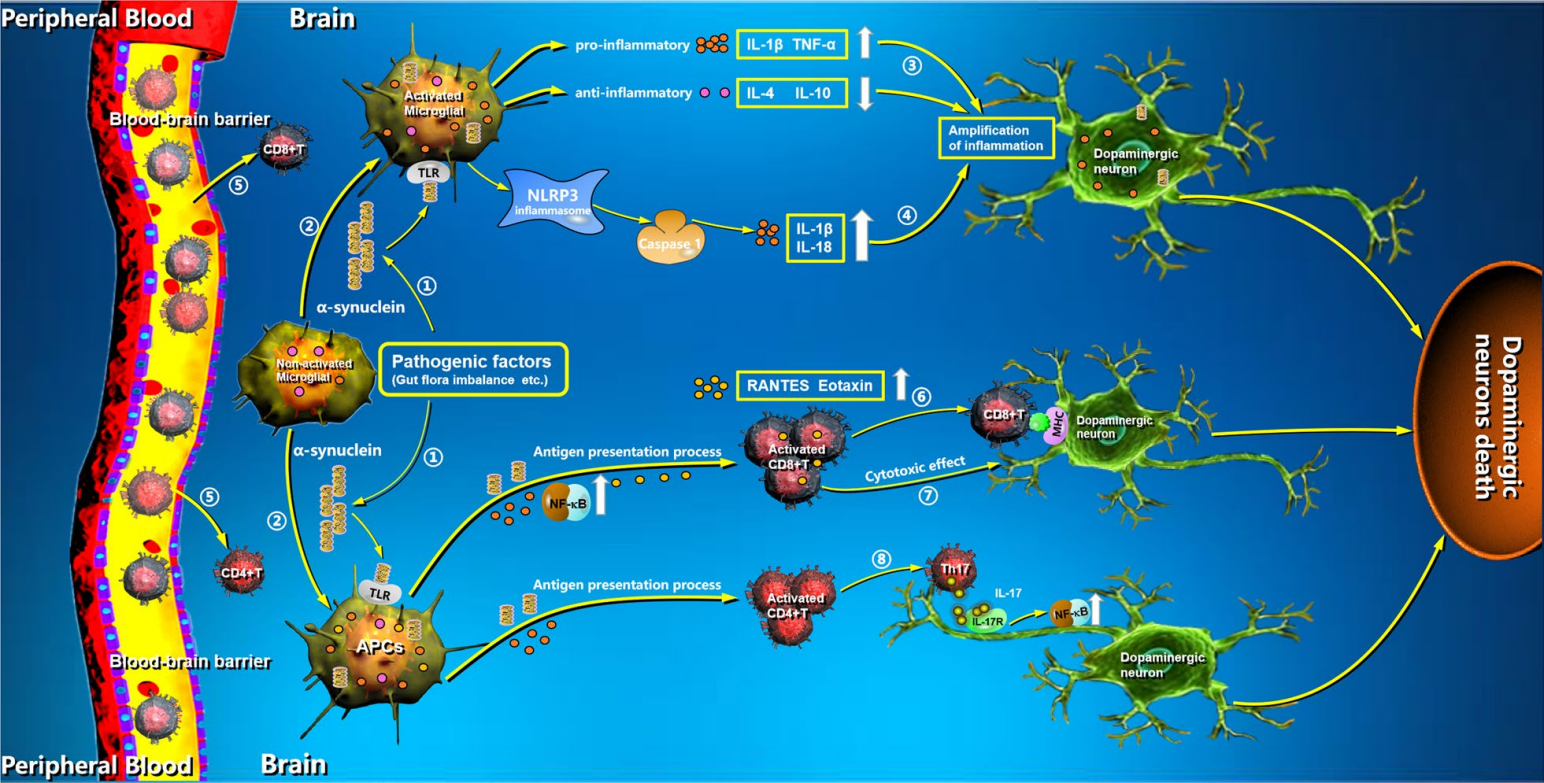
Mechanisms and roles of microglia activated by α-Syn on T cells in PD. Pathologic factors cause abnormal α-Syn accumulation in the brain parenchyma (1). Toll-like receptors on microglia can recognize misfolded α-Syn and promote the transformation of non-activated microglia into activated microglia and antigen-presenting cells (2). Proinflammatory cytokines and ROS secreted by microglia amplify neuroinflammatory responses in the brain and hasten the death of dopaminergic neurons (3). α-Syn aggregation in microglia activates NLRP3 inflammasomes, amplifying the inflammatory response and damaging dopaminergic neurons (4). The blood–brain barrier is compromised with age and disease, allowing peripheral blood immune components such as T cells to infiltrate the brain parenchyma (5). Activated microglia increase the secretion of inflammatory chemokines like RANTES and eotaxin, increasing CD8 + T cell infiltration and the cytotoxic response to dopaminergic neurons (6, 7). T lymphocyte-induced dopaminergic neuron death was mediated by the IL-17-IL-17R signaling pathway, which then activated the downstream NF-B signaling pathway (8). The images in this figure were downloaded from ProteinLounge.com with permission to use them

The gut flora and other microbes control integral functions of immune cells in the gut, periphery, and brain. The presence of intestinal inflammation or gastrointestinal abnormalities such as constipation and diarrhea often precedes movement disorders in PD patients for many years [31, 46]. According to Braak’s hypothesis, abnormal α-Syn accumulation begins in the gut and spreads to the brain through the vagus nerve. It appears early in the enteric nervous system, glossopharyngeal nerve, and vagus nerve. It is possible that shaving the vagus nerve has a reduced risk of PD (Fig. 1) [47]. The α-Syn travels in the brain in a prion-like fashion between different neurons and brain regions. Although there is no doubt that the gastrointestinal tract is involved in the occurrence and development of PD, there are still limitations in the study of PD and the brain-gut-microbe axis. Biopsy of the SN does not explain the timing of α-Syn deposition, systemic autopsy could not explain the dynamic process of α-Syn deposition, and animal experiments do not perfectly simulate the occurrence and development of PD. Even the mechanism of misfolding and deposition of α-Syn is not clear in vivo, so it is too early to directly implicate misfolding of α-Syn in the gastrointestinal tract as the origin of PD. Studies have found that α-Syn is expressed presynaptically in the perinuclear area in the central nervous system, and there is a dynamic balance between production and degradation in healthy people [48]. The ubiquitin proteasome system (UPS) and autophagy–lysosome pathway (ALP) are two critical intracellular mechanisms for removing misfolded or aged proteins, so they have a significant impact on maintaining α-Syn levels. The UPS selectively degrades intracellular, membrane, misfolded, and damaged proteins [49–51]. Parkin and UCHL1, with two genetic mutations found in PD, affect the function of the UPS, so α-Syn does not degrade effectively and forms Lewy bodies, and the ALP pathway is a compensatory degradation mode for the damaged UPS [52, 53]. Neuronal cells have only a limited ability to remove α-Syn, and misfolding and/or neuronal internalization of α-Syn promote conformational template formation of endogenous α-Syn monocytes by mechanisms similar to prion-like spreading [30].

### PD and microglial activation

Microglia, as the first line of defense of the immune system, similar to macrophages in the central nervous system, can respond quickly to invading pathogens, changes in the physiological microenvironment, and damage to the central nervous system, playing a centric role in the central inflammatory response [10, 54, 55]. Acute inflammatory reactions mediated by microglia are generally thought to benefit neuronal survival via the predominant phenotype M2. Nevertheless, the long-term and excessive activation of microglia can result in chronic inflammatory events, which unexpectedly exert a detrimental influence, as demonstrated in neurodegenerative diseases such as PD. When activated by pathological injury in the brain, microglial cells can rapidly change their morphological characteristics, increase their motility and phagocytic activity, and secrete cytokines that fight pathogens and promote tissue remodeling and repair [54]. Toll-like receptors (TLR) are a family of pattern-recognition receptors that recognize pathogen-related molecular patterns and misfolded proteins [56]. Microglia can recognize misfolded α-Syn by receptors such as TLR2 and TLR4 on the cell surface, so the binding of α-Syn to TLR2 and TLR4 can initiate related signaling pathways, such as NF-κB and the upregulation of proinflammatory cytokines and promote the transformation of anti-inflammatory microglial phenotype M2 into the proinflammatory microglial phenotype M1 [57]. The M2 phenotype of microglia can secrete anti-inflammatory factors IL-4, IL-10, and transforming growth factor-β (TGF-β), to significantly reduce and/or produce a large number of proinflammatory cytokines such as IL-1β, tumor necrosis factor-α (TNF-α) and reactive oxygen species (ROS) (Fig. 1) [57, 58]. Proinflammatory cytokines and ROS secreted by phenotypic M1 microglia further amplify neuroinflammatory responses in the brain and exacerbate the death of dopaminergic neurons [58].

### PD and pyroptosis

Microglia are the main cell mediators of brain inflammation during the inflammatory response. Inflammatory responses mediated by inflammatory cytokines such as IL-1β secreted by microglia play an essential role in the development and progression of PD [59]. The major events that regulate the secretion of IL-1β by microglia are the activation of inflammasomes and a programmed inflammatory cell death process called pyroptosis [60]. Pyroptosis is a new type of programmed cell death related to an inflammatory response with similar characteristics but different mechanisms than apoptosis [61]. It is not regulated by the apoptosis-related protein caspase-3, but rather depends on the regulation of the inflammation-related protein caspase-1 [62]. The NLRP3 inflammasome plays an important role in pyroptosis, which is a critical component of the innate immune system that mediates caspase-1 activation and the secretion of proinflammatory cytokines [63]. Inflammasomes are associated with some autoimmune diseases and neurodegenerative diseases such as PD [64]. Activation of NLRP3 promotes the secretion of inflammatory factors such as IL-1β and IL-18 and induces pyroptosis, which enhances inflammatory effects by promoting the secretion of IL-1β and activation of inflammasomes [64, 65]. There is also a close correlation between pyroptosis and α-Syn aggregation. The aggregation of α-Syn can be released from damaged neurons into the extracellular space to recognize TLR receptors on microglia, which activates the NF-κB pathway and promotes the production of the IL-1β precursor protein [57, 66]. The aggregation of α-Syn in microglia activates NLRP3 inflammasomes and induces pyroptosis as a danger signal [67]. Inflammatory cytokines IL-1β and IL-18 secreted outside the cells further amplify the inflammatory response and damage dopaminergic neurons (Fig. 1).

In summary, the neuroimmune response induced by excessive or misfolded α-Syn is likely to be caused by microglia-mediated inflammation. The misfolded and deposited α-Syn can activate the adaptive immune response, which induces the pathological development of PD. This novel mechanism may explain the pathogenesis of PD and offer a new strategy for treatment.

## Mechanisms and roles of microglia on T cells in PD

### PD and T cell

In 1988, P L McGeer et al. identified a unique pattern of HLA-DR-positive microglia by double immunostaining for glial fibrillary acidic protein and HLA-DR in PD patient samples obtained after death, which indicated that the disease might be related to a neuroimmune response [68]. Studies have also shown a decrease in the number of T cells, including CD4 + and CD8 + T cells in the peripheral blood of PD patients, the high expression of MHC molecules in mononuclear cells, and the detection of a large number of activated T cells in the cerebrospinal fluid [69–72]. This phenomenon indicated that the peripheral immune components might enter the central nervous system in PD patients and that the ability of peripheral blood T cells to enter the brain may be the result of systemic inflammatory responses. Studies have shown that the integrity of the BBB in the ventral midbrain and deep cortex is damaged due to inflammation in PD patients and as a result, T cells can infiltrate into the brain (Fig. 1) [70, 73–75]. Studies have also shown that the levels of CD4 + and CD8 + T cells in the SN of the brain are significantly higher in PD patients than that in healthy people [76–78]. It is worth noting that T cells are mainly distributed near blood vessels and neuromelanin-positive dopaminergic neurons, which suggests that T cells are likely to interact with dopaminergic neurons through targeted migration [75, 79].

### Microglia promote infiltration of CD8 + T cells

Roy et al. reported marked upregulation of RANTES (regulated on activation, normal T-cell expressed and secreted) and eotaxin, chemokines that are involved in T cell trafficking, in the serum of hemi-Parkinsonian monkeys [80]. The infiltration of CD8 + T cells around the hemi-Parkinsonian monkey’s parenchyma was significantly increased compared to that of the control group, which indicated that they were highly likely to migrate to the parenchyma by chemotaxis [80]. In addition, they discovered that in a 1-methyl-4-phenyl-1,2,3,6-tetrahydropyridine (MPTP) PD mouse model, the expression of RANTES and eotaxin was up-regulated in microglia cells of brain parenchyma, and confirmed that activation of the NF-κB signaling pathway is the most important regulatory pathway for inflammation in the upregulation of these two chemokines, because this pathway controlled the transcription and expression of proinflammatory factors such as RANTES and Eotaxin in many cells [81]. The NF-κB activation of microglia in the SN has been confirmed in MPTP-treated mice and monkeys and PD patients, and the role of NF-κB in the adaptive immune response of PD brain SN has been investigated [82–84]. These results suggest that activation of the NF-κB signaling pathway in microglia under pathological conditions of PD promotes the secretion of inflammatory chemokines such as RANTES and eotaxin, increasing the infiltration of CD8 + T cells into PD brain SN and the cytotoxic response to dopaminergic neurons (Fig. 1).

### Microglia regulate CD4 + T cells

In 2017, two studies showed that autoreactive T cells could recognize specific α-Syn epitopes in PD patients [85, 86]. Researchers observed infiltration of CD4 + Th17 cells was also observed in an MPTP mouse model and that Th17 cells increased the death of dopaminergic neurons in MPTP-treated ventral midbrain dopaminergic neuron cultures [87, 88]. In the same study, the death of midbrain neurons derived from autologous induced pluripotent stem cells in PD patients may be closely related to CD4 + Th17 cells secreting IL-17, indicating that in addition to the neuronal susceptibility to CD8 + cytotoxic T lymphocytes, CD4 + T cells are also involved in the pathological process of PD [88]. The death of T lymphocyte-induced dopaminergic neurons was found to be mediated through the IL-17-IL-17R signaling pathway, which further activated the downstream NF-κB signaling pathway associated with cellular inflammatory and immune responses (Fig. 1) [88]. Activated microglia can be used as antigen-presenting cells (APCs) to present autoantigens such as α-Syn by MHC molecules [85]. The T cells activated by α-Syn misfolded proteins mainly involve CD4 + T cells, which proliferate and release large amounts of proinflammatory cytokines, leading to the apoptosis of dopaminergic neurons [87]. In addition to the activation of T cells by APCs, another clinical study of PD also showed that neuroinflammatory conditions such as secretion of inflammatory cytokines can trigger the expression of MHC-I molecules on the surface of dopaminergic neurons, making them vulnerable to attacks by CD8 + T cells, which also strongly supports the participation of the autoimmune response in PD patients [85].

In summary, CD4 + /CD8 + T cells expressing α-Syn were found in the brains of PD patients, where α-Syn could be recognized as an antigen by APCs, such as microglia or monocytes. The APCs can present antigens to CD4 + and CD8 + T cells, induce their activation, and trigger an autoimmune response in the brain, leading to the death of dopaminergic neurons.

## Mechanisms and roles of T cells on microglial cells in PD

### T cells regulate the activation of microglia

The regulation of T cells on microglia is also critical in the brain pathogenesis of PD patients. Some researchers have suggested that T cells are essential for the upregulation of MHC-II molecules in microglia, and this is an important process of α-Syn activation of microglia in the SN, leading to the loss of dopamine neurons [89–91]. David et al. found that α-Syn alone may not be sufficient to cause significant death of dopaminergic neurons in the SN, while T cells can accelerate the death of dopaminergic neurons by up-regulating the expression of MHC-II molecules in microglia [90]. This study found that MHC-II overexpression in vivo accelerated α-Syn-induced microglial activation, and MHC-II knockout prevented α-Syn-induced microglial activation, antigen presentation, and dopaminergic neuron degeneration. Meena et al. evaluated the effect of T cells on the neurodegeneration of PD by injecting the adeno-associated virus (AAV) encoding human wild-type α-Syn into the SN of T-cell-deficient rats [92]. The results showed no significant increase in microglial activation in the T-cell deficit group, whereas the rats without T-cell deficiency showed a significant increase in MHC-II positive microglial activation and significant loss of dopaminergic neurons. The presence of CD4 + and CD8 + T cells in the SN was also observed after MHC-II expression in microglia and loss of dopaminergic neurons [93]. The MHC is a group of cell surface proteins divided into two major classes of I and II molecules, that plays a fundamental role in adaptive immunity [94, 95]. Under normal conditions, MHC-I molecules are involved in endogenous antigen presentation and are commonly expressed in almost all cells, while MHC-II molecules are involved in exogenous antigen presentation and are mainly expressed by APCs, such as monocytes, macrophages, and dendritic cells [94]. Genome-wide association studies (GWAS) showed that PD was associated with the haplotype of the MHC-II gene, and the expression level of MHC-II was significantly increased in PD samples [96]. In addition, MHC-II positive microglia were detected in the SN of PD patients, and their expression level increased with the severity of the disease [90]. Activated microglia can express MHC-II molecules to further activate T cells, which in turn up-regulate the expression of MHC-II molecules in microglia to accelerate the activation process [91].

### Homeostasis of Teffs and Tregs

**Fig. 2:**
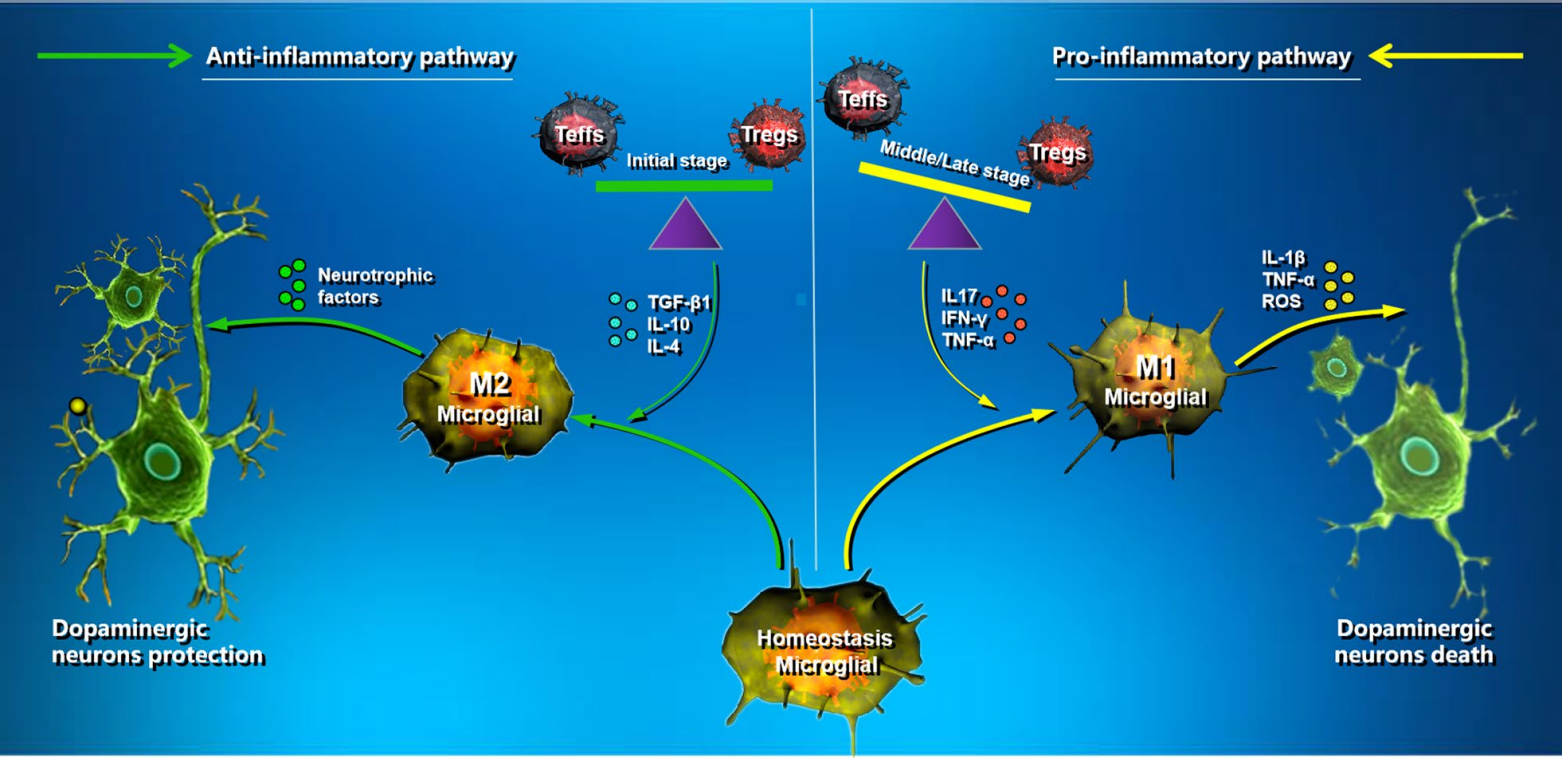
Mechanisms and roles of T cells on microglia cells in PD. The damaged BBB allows T lymphocytes, including Tregs, to enter the brain in the early stages of the disease, and Tregs can also play a role in immune regulation (left of the illustration). The immunosuppressive properties of Tregs gradually decrease as the disease progresses (middle and late stage), breaking the homeostasis between Tregs and Teffs. Teffs, in particular, hasten the activation of microglia cells by secreting various proinflammatory factors, which hastens the onset of neurodegeneration (right of the illustration). The images in this Figure were downloaded from ProteinLounge.com with permission to use them

It follows that T cells play a key role in the neuroimmune response of PD, but the regulatory effect of specific subtypes of T cells on microglia remains to be further explored. The T cells involved in the regulation of microglia in the neuroimmune system are classified as effector T cells (Teffs) and regulatory T cells (Tregs) and play a crucial role in maintaining neuroimmune homeostasis [97]. The balance of cellular immune function may control the rate of disease, and when this balance is disturbed, it can lead to disease. Studies have shown that CD4 + Teffs (such as Th1 and Th17) are involved in the process of neuron degeneration (such as in PD and MS) and maintain and accelerate the proinflammatory M1 phenotype of microglia by secreting proinflammatory phenotypic factors L17, IFN-γ, and granules B (Fig. 2) [45, 92, 98]. Similarly, in the MPTP model of PD, infiltrating T lymphocytes were found near the activated microglia and MHC-II was significantly overexpressed [92]. These MHC-II-overexpressed microglia further activated T lymphocytes through the antigen presentation process enhancing the immune response of Teffs to the neurodegenerative process.

### Tregs inhibit the activation of microglia

Tregs are naturally occurring subsets of CD4 + and CD25 + T lymphocytes [99]. In contrast to Teffs, anti-inflammatory phenotypes of cytokines secreted by Tregs have been shown to have neuroprotective effects. They are a major source of IL10 and TGF-β1 in vivo and are a major regulatory mechanism that controls innate and adaptive immune responses, so they have great potential to induce neuroprotection in PD [100]. Studies have shown that Tregs can inhibit microglial activation, promote neuronal survival in an MPTP-induced PD model, and are thought to inhibit the immune response through a variety of mechanisms [99, 100]. They also inhibit the functional metabolic pathway of Teffs through the release of anti-inflammatory factors such as IL-10 and TGF-β1. In addition to inhibiting the function and proliferation of Teffs, Tregs maintain anti-inflammatory microglia and astrocyte phenotypes by releasing IL-4, IL-10, and TGF-β (Fig. 2) [101, 102]. It has been shown that the transfer of CD4 + CD25 + Tregs to MPTP model mice with PD results in dose-dependent neuroprotective effects accompanied by inhibition of microglial activation, resulting in increased survival of dopaminergic neurons in the substantia nigra [99]. It has also been shown that Tregs can inhibit the function of M1-type microglia by inhibiting the activation of the NF-κB signaling pathway [91].

### PD and T cell homeostasis

The disruption of Teff and Treg homeostasis plays a crucial role in the neuroimmune response mechanism of PD [91]. In the initial stage of the disease, the damaged BBB allows T lymphocytes, including Tregs, to enter the brain and Tregs can also play a role in immune regulation at this stage. However, with the progression of the disease, the immunosuppressive properties of Tregs gradually decrease, which breaks the homeostasis between Tregs and Teffs. The Teffs then accelerate the activation of microglial cells by secreting various proinflammatory factors and further accelerate the occurrence of neurodegeneration (Fig. 2) [92].

### Summary of pathological mechanism

**Fig. 3:**
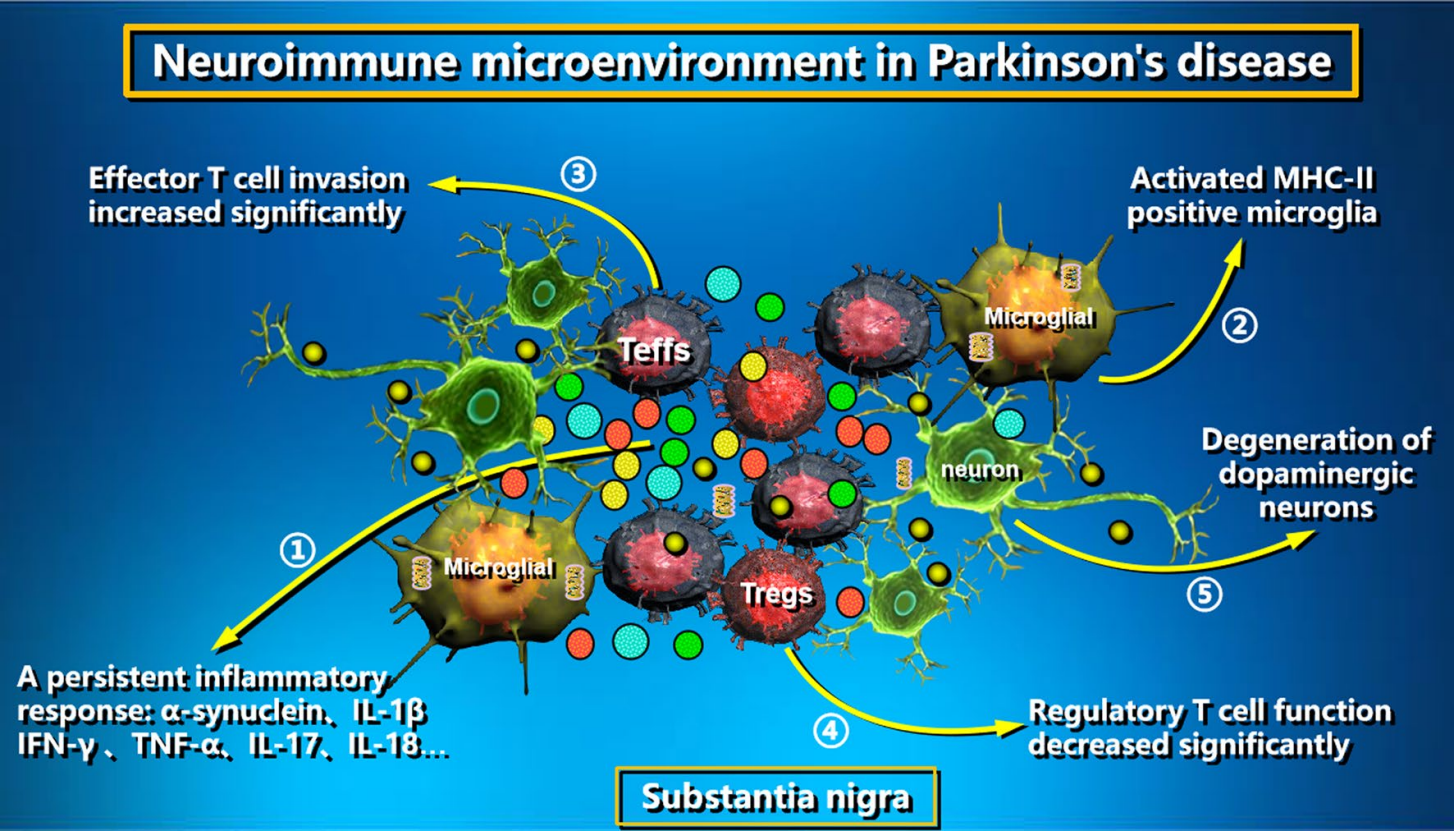
Neuroimmune microenvironment in PD. The neuroimmune microenvironment of PD is composed of several major factors, including a persistent inflammatory response (1), activated microglia (2), a balance disorder between Teffs and Tregs (3,4), and dopaminergic neurodegeneration (5), all of which contribute to the occurrence and progression of the disease through their interactive regulation. The images in this Figure were downloaded from ProteinLounge.com with permission to use them

Taken together, the major factors that constitute the neuroimmune microenvironment of PD include a persistent inflammatory response, activated microglia, a balance disorder between Teffs and Tregs, and degeneration of dopaminergic neurons, which leads to the occurrence and development of PD (Fig. 3). The α-Syn is the main pathological marker of PD. Under normal physiological conditions, there is a dynamic balance between the production and degradation of α-Syn in the human body, but the dynamic balance is broken and α-Syn is misfolded due to the action of various genetically or environmentally controlled pathological factors. Besides the abnormal α-Syn accumulation in the brain, the abnormal α-Syn accumulation starting in the gut can also be transmitted to the brain through the vagus nerve. During the course of the disease, the α-Syn can spread to different brain regions in a prion-like fashion. Microglia are the first immune defense system of the human brain and one of the main cell types involved in the inflammatory response of the central nervous system. Microglia can recognize misfolded α-Syn through TLR2 and TLR4 receptors on the cell surface and further initiate related signaling pathways, such as activating the NF-κB signaling pathway and up-regulating the expression of proinflammatory cytokines, to promote the transformation of anti-inflammatory phenotype M2 into proinflammatory phenotype M1. The activation of the NF-κB signaling pathway in microglia under pathological conditions of PD promotes the secretion of inflammatory chemokines such as RANTES and eotaxin, increasing the infiltration of CD8 + T cells in PD brain SN and the cytotoxic response to dopaminergic neurons. In addition, α-Syn can be taken up as an antigen by microglia and then acts as APCs, which in turn provide antigens to CD4 + and CD8 + T cells and cause them to activate and trigger an autoimmune response through multiple pathways, eventually leading to the apoptosis of dopaminergic neurons. The regulation of microglia by T cells invading the brain of PD patients is also an important form of pathogenesis. In the initial stage of the disease, the impaired BBB allows T lymphocytes, including Tregs, to enter the brain. As the disease progresses, the homeostasis between Tregs and Teffs is disrupted, and Teffs accelerate the activation process by secreting various pro-inflammatory factors to up-regulate the expression of MHC-II molecules in microglia. Moreover, further activation of microglia also increases their effect on T cells, and the interaction between the two kinds of cells creates a vicious cycle that accelerates mitochondrial dysfunction and apoptosis in dopaminergic neurons. Inhibiting autoimmune responses through external intervention such as stem cell therapy and increasing the number of Tregs in the brain is likely to become an important strategy for PD treatment.

### Immunomodulatory therapies targeting α-Syn for PD

A-Syn is the primary pathological marker of PD and the most promising therapeutic target for PD immunotherapy. Misfolded α-Syn accumulates in microglia and dopaminergic neurons, which can activate various immune pathways. As a result, targeting α-Syn to prevent intracellular aggregation and propagation is an important strategy for the prevention and treatment of Parkinson's disease. The current PD treatment focuses primarily on α-Syn clearance and degradation, as well as immunoregulation therapy for autoimmunity.

## A-Syn immunotherapy, both active and passive

### Active and passive

A-Syn forms oligomers and fibrils depending on its biological properties and the situation. However, it is subject to posttranslational modifications such as acetylation, phosphorylation, and truncation [103, 104], and it also targets related immune mediators primarily via an immune pathway to accelerate dopaminergic neurodegeneration [105, 106]. Immunotherapy, including active and passive immunity, is based on the principle of specific antigen/antibody binding [107, 108]. Active immunity is the use of antigens to stimulate the body's production of antibodies rather than directly introducing antibodies from outside the body, which has a high resistance to subsequent infection, either through the disease pathogen itself or through immunization [109]. Data show that naturally occurring anti-α-Syn autoantibodies help with α-Syn clearance, reduce protein aggregation, and protect neurons [107, 110]. Active immunization against PD has been tested in animal models and early clinical trials in a large population, and it has proven to be less expensive than other treatments [107, 111]. Vaccinations do not require frequent injections, which can relieve financial and psychological stress on patients. Active immunity is more suitable for patients with early Parkinson's disease and high-risk groups because it relies on the use of specific antigens that can produce inflammatory response antibodies [112, 113]. Passive immunity is the specific immunity acquired by the body by passively receiving antibodies, sensitized lymphocytes, or their products. Antitoxin, placental globulin, antibacterial serum, and immune modulators are common biological products of artificial passive immunity [100, 114]. Passive immunization is thus appropriate for patients with moderate to severe PD.

### A-Syn antibody preparations and clinical trials

Several studies have shown that an α-Syn transgenic mouse line, 83Vle/J, expressing human A53T variant α-Syn (the full-length, 140 amino acid isoform), can produce high-affinity antibodies and reduce α-Syn accumulation in dopaminergic neurons, thereby reducing neuronal damage [115, 116]. Furthermore, DNA vaccines can induce active immunity in the brain by inducing the production of antibodies that recognize misfolded α-Syn [117]. These peptide fragments, as well as the full-length recombinant α-Syn, were used to sensitize mouse dendritic cells, which were then intravenously delivered into transgenic mice expressing the human A53T variant of α-Syn, which could effectively produce specific antibodies and improve neuromotor function in vitro [116, 118]. Two short peptide vaccines (PD01A and PD03A) are being tested in clinical trials to assess the efficacy, tolerability, and safety of active immunization in Parkinson's disease patients (Table 1) [15, 16, 119]. Traditional antibodies, such as monoclonal (mAb) and polyclonal (pAb), have been at the forefront of biomedical research for diagnostic and analytical therapies against cancer, immune diseases, and infectious diseases [120]. They do, however, have limitations, such as stability over narrow pH and temperature ranges and the potential inability to access specific active sites on proteins [120]. Single-stranded antibodies have superior tissue penetration, the ability to cross the blood–brain barrier, the unique ability to bind to small cavities or clefts, high affinity and specificity, and high stability at extreme temperatures and pH [120, 121]. These advantages of single-stranded antibodies can improve their therapeutic effect and be used to treat PD [121]. A clinical trial of antibody preparation MEDI1341 was conducted to reduce extracellular α-Syn levels in healthy volunteers, and the study evaluated safety and tolerability (Table 1) [122]. MEDI1341 rapidly enters the central nervous system after intravenous injection into rats and cynomolgus monkeys, lowering free extracellular α-Syn levels in the interstitial fluid (ISF) and cerebrospinal fluid (CSF) compartments [123]. AstraZeneca and Takeda are collaborating to develop the antibody MEDI1341, which reportedly retains reduced immune effector function [122]. Clinical trials of BIIB054 and PRX002, which directly target the amino terminus of α-Syn and are highly selective for the aggregation form of α-Syn (Table 1), have also been conducted [17, 124, 125]. BIIB054 binds to α-Syn in PD and DLB tissue sections and extracts but not in unaffected brains and prevents dopamine transporter reduction induced by injection of preformed α-Syn fibrils into mouse brains [122]. This study found no evidence of clinical efficacy. Each of the three infusions resulted in significant reductions in free serum α-Syn levels within 1 h of PRX002 administration. PRX002 levels in serum and CSF increased in a dose-dependent manner [125]. These studies looked at the efficacy, safety, tolerability, pharmacokinetics, and pharmacodynamics of PD patients.

**Table 1:** Clinical trials of immunotherapy against α-Syn in PD

Compound	Trial ID/phase	Mechanism	Test purpose
*Active immunotherapy*			
PD01A	NCT01568099/I	A short epitope peptide that induces the production of antibodies with a high affinity to α-Syn	Evaluation of efficacy, tolerability, and safety of active immunization in PD patients
	NCT02270489/I		
PD03A	NCT02267434/I	A vaccine that targets α-Syn and induced antibodies differ in specificity to PD01A	
	NCT02270489/I		
*Passive immunotherapy*			
MEDI1341	NCT03272165/I	Antibody preparation which has been conducted to reduce extracellular α-Syn levels	Evaluation of safety and tolerability on healthy volunteers
BIIB054	NCT02459886/I	Antibody preparation which directly targets the amino terminus of α-Syn and is highly selective for the aggregation form of α-Syn	Assessment of the safety, tolerability, pharmacokinetics, and pharmacodynamics of PD patients
PRX002	NCT02157714/I		

## MSCs modulate α-Syn in PD: a multitarget disease-modifying therapies strategy

### MSCs and neuroinflammatory disease

MSCs, as adult stem cells capable of paracrine, immune regulation, and multidirectional differentiation, have several advantages over embryonic stem cells, including a lower immune response, a lower risk of tumor formation, and no ethical concerns [126–129]. More research has been done on the relationship between MSCs and inflammatory processes since the discovery of their immunomodulatory properties. There is evidence that MSCs can migrate to the site of an inflammatory response via chemotaxis and have an immunomodulatory effect on specific chemotaxis recruitment responses, thereby reducing the inflammatory response in the damaged area and promoting tissue repair [130–132]. MSC-based cell therapies have been used to treat a variety of autoimmune-related neurodegenerative and neuroinflammatory diseases, including PD, Alzheimer's disease, multiple system atrophy, and amyotrophic lateral sclerosis [133–136]. The main pathogenic mechanism in PD is thought to be the loss of dopaminergic neurons, and most researchers believe that the therapeutic effect is due to MSCs' cell replacement ability. MSC research has shifted in recent years from cell replacement to multitarget therapy such as paracrine and immune regulation (Table 2).

**Table 2:** multitarget disease-modifying therapies of MSCs modulates α-Syn in PD

Compound	Cell source	Mechanism	Study description and purpose
Immunomodulation	Bone marrow	MSCs can promote the polarization of M2 phenotype microglia and reduce the aggregation of α-Syn in cortical areas by activating IL-4-related STAT signaling pathways	MSCs exert a neuroprotective effect via the clearance of extracellular α-synuclein by controlling microglia M2 polarization, suggesting that MSCs could be used as a disease-modifying therapy for patients with α-synucleinopathies
	Bone marrow	MSCs can modulate microglia activation through TSG-6 and that TSG-6 attenuates the inflammatory cascade in activated microglia	The immunomodulatory effect of MSCs on microglia and that MSCs might be promising therapeutic agents for the treatment of neurotraumatic injuries or neuroinflammatory diseases associated with microglial activation
Autophagy	Bone marrow	MSCs can exert neuroprotective effects through enhancement of autophagolysosome formation and modulation of a-synuclein expression, possibly via the autophagic pathway	The modulation of the autophagic pathway to control PD-related microenvironments using MSCs will have a significant impact on future PD treatment strategies
Mediate endocytosis	Human MSCs (Obtained from the Severance Hospital Cell Therapy Center)	MSCs can block extracellular α-Syn clathrin-mediated endocytosis (CME) by regulating the interaction with n-methyl-d-aspartic acid (NMDA) receptors	MSCs can exert neuroprotective properties through inhibition of extracellular α-synuclein transmission, suggesting that the property of MSCs may act as a disease-modifying therapy in subjects with α-synucleinopathies
Bioprotease	Human MSCs (Obtained from the Severance Hospital Cell Therapy Center)	MSCs secreted MMP-2 molecules can disrupt newly formed amyloid deposition, significantly reducing α-Syn insolubility and oligomeric levels	MSCs can exert neuroprotective properties through proteolysis of aggregated α‐synuclein into soluble forms, and have a significant impact on future PD treatment strategies

### Toxin-based animal models

PD is a multifactorial disease caused by genetic and environmental factors. Toxin-based animal models (such as 6-OHDA and MPTP) have primarily aided in the development of treatments for motor symptoms. Toxin-based models have advantages in that the symptoms are clear, the experiment period is short, they can be applied to a wide range of animal species, they show prodromal symptoms, and they will be used in future models [137]. According to Roy A et al., T-cell migration-related cytokines RANTES and eotaxin were up-regulated in microglia in the brain parenchyma of MPTP monkey models, while CD8 + T cell infiltration around the deep brain parenchyma of PD monkeys was significantly increased [80]. This study also showed that MPTP animal models could simulate some pathological processes in PD patients.

### Transgenic animal models

The α-Syn gene, the causative gene of familial PD, is one of the most important genetic factors in PD patients, and many α-Syn transgenic animal models have been created, contributing to the elucidation of PD pathology [137, 138]. The α-Syn transgenic model can replicate α-Syn aggregation and show slowly progressive changes, similar to human PD. Furthermore, injected α-Syn fibrils have been shown in mouse experiments to spread throughout the brain [139]. The propagation model created by exogenously administered α-Syn fibrils is still developing, but obtaining a phenotype is relatively simple, and the experiment takes less time than the transgenic model [139, 140]. The fact that this model can be applied to primates and other animals is a huge plus.

According to one study, exosomes isolated from MSCs can significantly improve the motor, learning, and memory abilities of the progressive in α-Syn A53T transgenic mice model, and the mechanism may be related to changes in phospholipid composition and cholesterol metabolism in hippocampal neurons [141]. Currently, the use of MSCs in transgenic mouse models is relatively rare, with most studies focusing on toxin-based models. As a result, we will investigate the role and mechanism of MSC intervention in MPTP toxicity and α-Syn-induced cell models.

### Modulating microglia activation

Microglia cells are the most active immune cells in brain tissue, and their mediated inflammatory response is linked to the pathogenesis of many immune-related nervous system diseases. Changes in the extracellular microenvironment can cause microglia to polarize into two cell phenotypes, M1 and M2. Misfolded α-Syn, though toxic to dopaminergic neurons, can activate microglia cells, causing them to polarize to the M1 phenotype, amplifying the neuroinflammatory response and secreting numerous proinflammatory factors [144, 145]. Because the phenotype mediated by α-Syn is involved in the pathogenesis of PD, external intervention to promote the polarization of microglia toward the M2 phenotype has important clinical value in the neuroinflammatory response [146, 147]. Lee et al. demonstrated that MSCs could induce M2 phenotype microglia cell polarization, significantly improve phagocytic clearance of α-Syn in microglial cells, and have a significant neuroprotective effect in α-Syn-rich cells and animal models [26, 28, 146]. In the same study, the activated recombinant α-Syn protein was injected unilaterally into the right neocortex of mice. The findings confirmed that MSCs could promote the polarization of M2 phenotype microglia and reduce α-Syn aggregation in cortical areas by activating IL-4-related STAT signaling pathways in microglia cells and α-Syn inoculated IL-4 KO mice [146]. Bidirectional studies using siIL-4 MSCs in α-Syn mice and MSCs in IL-4R-KO mice confirmed that they could counteract α-Syn phagocytosis of microglia cells by promoting M1 polarization [146]. They discovered that cerebrospinal fluid (CSF) from MSC-transplanted multiple system atrophy patients induced microglia M2 polarization and had a prosurvival effect in α-Syn-treated BV2 cells via enhanced clearance of α-Syn [146]. Another study found that MSCs could regulate microglial cell activation and reduce the inflammatory cascade reaction of activated microglia cells via TNF- stimulated gene/protein 6 (TSG-6), implying that MSCs and TSG-6 could be effective ways to treat neuroinflammatory diseases associated with microglial cell activation [148].

### Enhancing autophagy

**Fig. 4:**
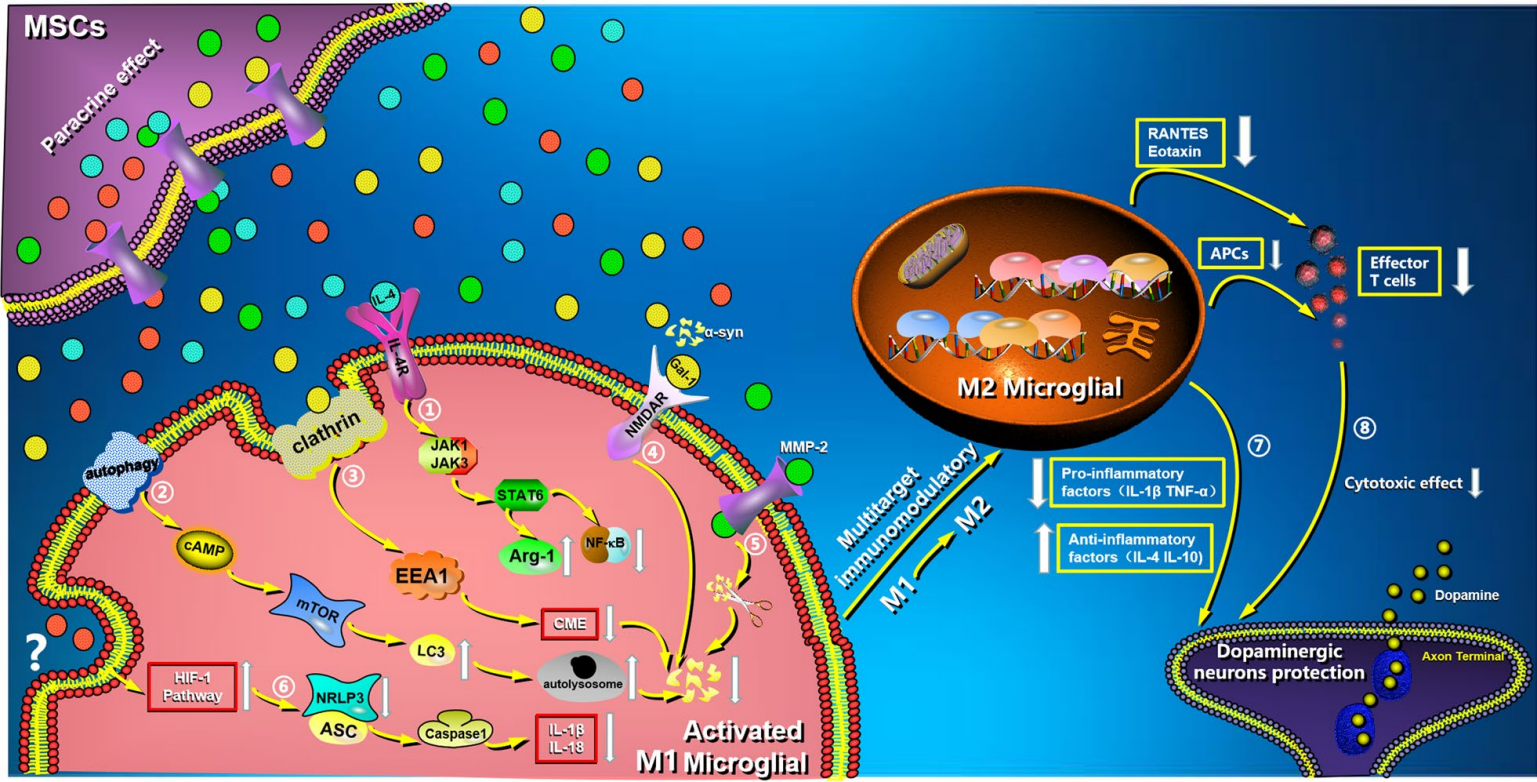
Mechanisms of MSC multitarget immunotherapy in PD. Through their paracrine effect, MSCs secrete a large number of immunomodulatory cytokines. MSCs can promote M2 phenotype microglia polarization and reduce α-Syn aggregation in cortical areas by activating IL-4-related STAT signaling pathways (1). MSCs can significantly increase the formation of LC3-positive autophagosomes and decrease α-Syn aggregation (2) by activating the autophagy signaling pathway. MSCs inhibit extracellular α-Syn CME by regulating clathrin and EEA1, as well as the interaction between α-Syn and nNMDA receptors by releasing Gal-1, which completely binds NMDA receptors with α-Syn (3, 4). MSCs can eliminate abnormal α-Syn expression and accelerate its degradation via secretory small molecule cleavage, such as MMP-2 (5). We hypothesized that hypoxia preconditioning MSCs would prevent pyroptosis in microglia cells by down-regulating the NLRP3 inflammasome via the HIF-1 signaling pathway (6). MSCs can significantly induce M2 phenotype microglia cell polarization and have a significant dopaminergic neuronal protective effect by attenuating inflammation amplification (7). MSCs can significantly induce M2 phenotype microglia cell polarization and have a significant dopaminergic neuronal protective effect by attenuating the antigen presentation process and T cell chemokine secretion (8)

Autophagy is the primary pathway for the degradation of harmful aggregation proteins in a variety of neurodegenerative diseases. Autophagy has been shown to play an important role in the pathogenesis of PD, and autophagy disorders can result in abnormal α-Syn aggregation, which can be cleared by increased autophagy [27, 149]. This suggests that autophagy regulation could be used to treat PD. The PD models exhibited increased autophagy and neuronal apoptosis, owing to the failure of most autophagosomes to fuse with lysosomes as a result of α-Syn misfolding and the resulting neuroinflammatory response [150–153]. MSCs have been shown in studies to significantly increase autophagosome formation and reduce α-Syn aggregation after intervention in PD models, and the neuroprotective effect of MSCs is largely dependent on lysosomal activity mediated by autophagosome formation [154, 155]. Park et al. used human bone marrow-derived MSCs to interfere with an MPTP-treated neuron cell model, which significantly reduced neuron apoptosis and abnormal α-Syn expression while increasing the number of LC3-positive autophagosomes (Fig. 4) [154]. MSCs derived from human bone marrow were injected into the tail vein of MPTP-treated PD animal models, which can also significantly enhance the maturation of late autophagic vacuoles and fuse with lysosomes to form autophagosomes [154]. These findings suggest that MSC therapy could significantly improve autophagosome formation and α-Syn clearance in PD models.

### Mediating endocytosis

Extracellular α-Syn aggregates' prion-like behavior is important in the pathogenesis and progression of α-Synucleinopathies [156–158]. Although the precise mechanism of α-Syn intercellular transmission is unknown, receptor-mediated endocytosis is likely to be an important route for α-Syn into cells. MSCs have been shown to inhibit extracellular α-Syn clathrin-mediated endocytosis (CME) by modulating interactions with n-methyl-d-aspartic acid (NMDA) receptors [26]. MSCs can inhibit extracellular α-Syn CME by regulating clathrin and early endoplasmic reticulum antigen 1 (EEA1), as well as the interaction between α-Syn and the NMDA receptor in α-Syn aggregated cells (Fig. 4) [26]. MSCs were also found to reduce α-Syn endocytosis by releasing galectin-1 (Gal-1) to competitively bind NMDA receptors with α-Syn [26].

### Protease secretion

Because protease can cut abnormal α-Syn aggregates, using an exogenous protease's cutting function may be able to clear abnormal α-Syn aggregation in PD. MSCs have been shown to secrete molecular substances into the nervous system microenvironment to promote proteolysis. MMP-2 is a soluble factor derived from MSCs that can participate in the hydrolysis of α-Syn proteins [28, 159]. MMP-2 molecules can disrupt newly formed amyloid deposition, reducing α-Syn insolubility and oligomeric levels significantly (Fig. 4) [28]. MSCs can thus eliminate abnormal α-Syn expression and accelerate its degradation via their small secretory molecule.

### The hypothesis of inhibiting pyroptosis

MSC therapy for PD associated with microglia pyroptosis has not yet been studied. In vitro, hypoxia-preconditioned olfactory mucosal mesenchymal stem cells (OM-MSC) inhibit pyroptotic death of microglial cells in response to cerebral ischemia/reperfusion injury by activating hypoxia-inducible factor-1 (HIF-1) [160]. The activation of the NLRP3 inflammasome in BV2 microglia cells exposed to ischemia/reperfusion injury was investigated in this study, and it was discovered that the expression of ASC, caspase 1, caspase 8, and GSDMD proteins was increased after ischemia/reperfusion injury. Furthermore, IL-1 and 1L-18 levels increased following injury. It was also shown that co-culture with hypoxia preconditioned OM-MSCs significantly reduced pyroptotic cell death in microglia following cerebral ischemia/reperfusion injury compared to normal OM-MSCs. In addition, α-Syn aggregation in microglia activates NLRP3 inflammasomes and induces pyroptosis as a danger signal, causing microglia to switch from anti-inflammatory phenotype M2 to pro-inflammatory phenotype M1 [67]. IL-1 and IL-18 as two inflammatory cytokines produced extracellularly increase the inflammatory response and destroy dopaminergic neurons. Furthermore, hypoxia-preconditioned OM-MSCs dramatically reduce IL-1 and IL-18 production in microglia, indicating that OM-MSCs may prevent pyroptosis in microglia. It was hypothesized that hypoxia preconditioning of MSCs may prevent pyroptosis of microglia cells in neurodegenerative diseases associated with neuroinflammatory responses, such as PD (Fig. 4).

### Summary of multitarget disease-modifying therapies

Taken together, dopaminergic neuron degeneration is caused by the interaction of α-Syn, microglia, and T cells during the course of PD. As a result, if one or more of these links can be targeted to slow disease progression, it will be an important strategy for PD prevention and treatment. In summary, the use of MSCs can accelerate the clearance and degradation of α-Syn via multitarget disease-modifying therapies mechanisms such as modulating microglia activation, enhancing autophagy, mediating endocytosis, protease secretion, and possibly preventing pyroptosis of microglia. Furthermore, one study found that MSCs inhibit the differentiation of CD4 + T cells into Th17 cells isolated from PD patients and that this suppressive effect was primarily associated with an increase in functional CD25 + Foxp3 + Treg cells and IL-10 secretion [161]. Furthermore, studies have shown that MSCs can be induced to differentiate into dopaminergic neurons and improve neural function by replacing lost neurons in the brain of people with PD. MSCs as cell-based therapy candidates in PD have a broader scope than simple cell type replacement because they can be used as a cellular system for the detoxification of reactive oxygen species (ROS) as well as a supplier of neurotrophic factors via paracrine effect to protect the mitochondrial function of dopaminergic neurons [162]. Based on progress in understanding MSCs' multitarget disease-modifying therapies effect, we believe MSCs could be used as an important strategy for therapy or to prevent the onset of PD in at-risk individuals, as well as to slow the progression of the disease.

## Major challenges of MSC-based cell therapy

### Allogeneic and autologous MSCs

Although MSCs have tremendous potential and opportunities in the treatment of immune diseases such as PD, their clinical application faces numerous challenges. The differences in cell sources and amplification schemes, as well as the decreased survival rate, paracrine effect, and homing ability caused by the local adverse microenvironment following cell transplantation, limit MSCs' therapeutic effect. MSCs derived from allogenic sources have received the most attention and application over the last few decades. Despite numerous reports describing MSC immune privilege or low immunogenicity, other studies have shown that allogeneic MSCs enhance inflammation and are subsequently cleared in response to rejection by donor-specific antibody levels, suggesting that this upregulation may be the reason for allogeneic MSC treatment failure [163, 164]. Interestingly, one study found that the immunogenicity of MSCs depended on whether they differentiated in vivo, with undifferentiated MSCs having low immunogenicity, while MSCs after differentiation had significantly higher immunogenicity [165]. These factors also reduce MSCs' therapeutic effect to some extent [163, 165]. Although allogeneic MSCs may be immunogenic, their potent immunomodulatory effects have opened up new avenues for immunomodulatory inflammatory diseases and attracted researchers in related fields. Researchers discovered in 2010 that OM-MSCs isolated from human nasal mucosa were a better source of MSCs [166]. Since 2014, our research team has been studying autologous human OM-MSCs and has developed a complete culture system. These cells have a higher rate of proliferation and a shorter passage time. OM-MSCs are widely distributed in the nasal cavity, are easily accessible, exhibit no immune rejection, have few or no associated ethical issues [127, 167], and have the added benefit of retaining biological activity that does not change with age [168]. As previously stated, nasal mucosa develops from embryonic ectoderm and can directly differentiate into neurons under the right conditions. According to chromosome karyotype analysis and tumor gene analysis, there is no genetic variation after substantial in vitro passages [169]. It is unknown, however, whether the success of autologous MSC transplantation is impacted by pathologic genetic background since there have been no reports of clinical studies using autologous MSC transplantation for the treatment of neurodegenerative diseases. Recently, we confirmed that OM-MSCs transplantation could alleviate the symptoms of Alzheimer's disease in APPswe/PS1dE9 mice and promote Aβ clearance through immunomodulation, thus demonstrating the great potential and social value of OM-MSC treatment for Alzheimer's disease patients. What's more, our findings confirmed that OM-MSCs could differentiate in multiple directions, including neurons, bone tissue, and adipose tissue [170]. Thus, the benefits of OM-MSCs make them an ideal source of cells for treating neuroimmune diseases such as PD.

### Challenges and improvements for MSCs

The cell survival rate, number of homing cells, and immunomodulatory effects of MSCs after implantation in vivo are critical to the success of MSCs therapy. Excessive inflammatory response, oxidative stress, hypoxia, and other negative factors in the microenvironment at the site of injury of the inflammatory immune disease may be the most important factors limiting MSC survival and efficacy. During in vitro amplification, these MSCs were cultured in 21% oxygen and 10–20% serum concentration, but after implantation in disease models or patients, they were exposed to hypoxic or ischemic microenvironments. In this case, MSCs rely solely on anaerobic glycolysis activation to obtain weak energy and may undergo self-apoptosis [171]. Many studies have shown that pretreatment with hypoxia, hypertrophic medium, cytokines, or chemicals can improve MSC survival, homing ability, and paracrine effect after transplantation [172, 173]. The primary goal of MSC pretreatment is to allow them to adapt to the harsh local microenvironment ahead of time while also increasing their survival rate, paracrine, immune regulation, and differentiation functions. Previous research has shown that a hypoxic microenvironment can promote OM-MSC proliferation and differentiation into dopaminergic neurons [128]. In vitro, hypoxia-preconditioned OM-MSC inhibited pyroptotic death of microglial cells in response to cerebral ischemia–reperfusion insult by activating hypoxia-inducible factor-1 [160]. Hypoxic pretreatment has also been shown in studies to improve MSC migration and homing ability and increase the number of damaged local MSCs [174, 175]. Furthermore, the immunomodulatory effects of MSCs are mediated by a cascade of inflammatory cytokines at the site of implantation [176], and preconditioning MSCs with inflammatory cytokines can increase the expression of intercellular adhesion molecules, inducible oxide synthase (iNOS), and chemokines while inhibiting T cell proliferation [177, 178]. The optimal culture scheme for MSCs to improve their survival rate, homing ability, and paracrine effect is a promising field of research that will address some of the challenges associated with MSC therapy. Finally, while MSC therapy has demonstrated pre-clinical success in treating various diseases, many other issues have been discovered during clinical trials. There is currently no international standard for the clinical application of MSCs, including cell dose, administration interval time, culture conditions, and route of administration. More research on the effects of the factors above and the biological characteristics of MSCs derived from various sources is required to find the best treatment for the various diseases and the potential for MSC-based therapies.

## Conclusions

In summary, α-Syn is the primary pathological marker of Parkinson's disease, and it can accumulate in the SN, triggering a neuroinflammatory response by activating microglia. This marker can further activate the neuroimmune response of dopaminergic neurons, which is mediated by reactive T cells via antigen presentation. The main components of the PD neuroimmune microenvironment are a persistent inflammatory response, activated microglia, a balance disorder between Teffs and Tregs, and dopaminergic neurodegeneration, which interact to cause the occurrence and progression of PD. MSCs can help to reduce the burden of toxic aα-Syn by promoting the M2 phenotype of microglia, increasing autophagy, increasing proteolysis of α-Syn aggregates, and inhibiting α-Syn intercellular transmission, allowing treatment via multitarget dominant immune regulation. Abnormal protein expression can be found in a variety of diseases with varying phenotypes, implying that abnormal α-Syn expression is a by-product of other major pathogenic pathways or immunomodulatory processes. Multitarget disease-modifying therapies involving neurodegenerative disease strategies are critical for the clinical efficacy of diseases characterized by abnormal protein expression, such as α-Syn, and the use of MSCs could be the most promising candidate for future treatment strategies.

## Data Availability

Not applicable.

## Abbreviations

PD: Parkinson’s disease
SN: Substantia nigra
A-syn: Alpha-synuclein
MSCs: Mesenchymal stem cells
BBB: Blood–brain barrier
UPS: Ubiquitin proteasome system
ALP: Autosomal system
TLR: Toll-like receptors
TGF-β: Transforming growth factor-β
TNF-α: Tumor necrosis factor-α
ROS: Reactive oxygen species
NLRP3: NLR family, pyrin domain-containing protein 3
ASC: Apoptosis-related speck protein containing the caspase activation recruitment domain
MHC: Major histocompatibility complex
RANTES: Regulated on activation, normal T-cell expressed and secreted
MPTP: 1-Methyl-4-phenyl-1,2,3,6-tetrahydropyridine
APCs: Antigen-presenting cells
AD: Alzheimer’s disease
MSA: Multiple system atrophy
ALS: Amyotrophic lateral sclerosis
CSF: Cerebrospinal fluid
TSG-6: TNF-α-stimulated gene/protein 6
LC3: Microtubule-associated protein 1 light chain 3
CME: Clathrin-mediated endocytosis
EEA1: Early endoplasmic reticulum antigen 1
NMDA: N-methyl-d-aspartic acid
Gal-1: Galectin-1
MMP-2: Matrix metalloproteinase-2
OM-MSCs: Olfactory mucosal mesenchymal stem cells
HIF-1α: Hypoxia-inducible factor-1α
